# Immune Defences: A View from the Side of the Essential Oils

**DOI:** 10.3390/molecules28010435

**Published:** 2023-01-03

**Authors:** Vivian Tullio, Janira Roana, Lorenza Cavallo, Narcisa Mandras

**Affiliations:** Department Public Health and Pediatrics, Microbiology Division, University of Torino, 10126 Turin, Italy

**Keywords:** essential oils, antifungal activity, yeasts, immune system, PMNs, intracellular killing

## Abstract

The use of essential oils is increasingly being investigated among new therapeutic approaches based on medicinal plants and their extracts. With the wide use of synthetic and semi-synthetic antimicrobial drugs, the spread of drug-resistant clinical isolates has increased, and research is directed towards natural products, such as essential oils, as useful antimicrobial resources. In the context of a prospective infection, we compared the impact of essential oils and common antimicrobial agents on the microbicidal activity of human phagocytes. Here, we present the results of our decades-long investigation into the effectiveness of thyme red oil (26.52% thymol chemotype), tea tree oil (TTO), and Mentha of Pancalieri [(*Mentha x piperita* (Huds) var. *officinalis* (Sole), form *rubescens* (Camus) (*Lamiaceae*)] essential oils on human polymorphonuclear leukocytes (PMNs) capacity to kill clinical strains of *Candida albicans* and *C. krusei* when compared to three antifungal drugs used to treat candidiasis (fluconazole, anidulafungin, and caspofungin) These essential oils demonstrate antifungal drug-like and/or superior efficacy in enhancing intracellular killing by PMNs, even at subinhibitory concentrations. Our results are compared with data in the literature on essential oils and immune system interactions. This comparison would aid in identifying therapeutic solutions to the increasingly prevalent antibiotic resistance as well as filling in any remaining knowledge gaps on the bioactivity of essential oils.

## 1. Introduction

In the last thirty years, there has been a significant increase in clinical interest in “natural” medicine, with a particular emphasis on the widespread use of plant products in the microbiological field. The increased resistance of microorganisms to antimicrobial drugs, and the emergence of newer diseases, require the urgent development of new, more effective drugs. Plants, due to the great biological and structural diversity of their components, can constitute a unique and renewable source for the discovery of new antimicrobial compounds. The use of essential oils is appealing and increasingly being investigated among new therapeutic approaches based on medicinal plants and their extracts [1,2]. Aromatherapy, a branch of alternative medicine that claims essential oils and other aromatic compounds have curative properties, has revived interest in essential oils in recent decades. Essential oils are concentrated hydrophobic liquids derived from plant secondary metabolism that contain volatile aroma compounds. Although they are widely employed in traditional medicine, the fragrance industry, and food flavoring and preservation, their good antimicrobial significance has only lately been recognized [3,4,5,6]. This new trend is due to the spread of microorganisms that are resistant to conventional antimicrobial agents, as well due to increased research into the antimicrobial activity of essential oils [1,7,8]. Drug resistance can arise because of bacteria and yeasts’ rapid “mutation”, which alters cellular membrane proteins in a way that prevents drugs from recognizing them [9]. In fact, most bacteria have the ability to alter membrane-associated macromolecules, the penicillin-binding proteins (PBPs), and enzymes that play key roles in the biosynthesis of the main component of the cell wall, the peptidoglycan. PBPs are the primary targets of β-lactam antibiotics. Many bacteria have the ability to produce a wide range of enzymes, including endo- and eso-β-lactamases or transferases, which can inactivate β-lactam and aminoglycoside antibiotics, respectively [10]. Efflux pump modifications, or a significant decline (by 50%) in ergosterol levels in fungal cellular membranes, which can no longer be the drugs’ target site, might cause yeasts to become resistant to antifungal treatments, particularly azoles [11]. In addition to these issues, antibiotics used to treat pathogen microorganisms may cause severe side effects, particularly in patients undergoing prolonged therapeutic treatment, or may alter the microbiome, which is critical for intestinal and overall body eubiosis. Antimicrobial agents, by increasing dysbiosis, create an ideal environment for pathogen microorganism colonization and subsequent infections, with the possibility of recurrent episodes. Therefore, with the wide use of synthetic and semi-synthetic antimicrobial drugs, the spread of drug-resistant clinical isolates has increased, and research is increasingly oriented towards natural products, such as essential oils, as useful antimicrobial resources [1,4,6,12]. Essential oils, on the other hand, contain hundreds of naturally active ingredients in varying proportions, which eliminates the risk of antibiotic resistance because microbes cannot adapt to their heterogeneous structure. Although the literature is fragmentary and incomplete, there is evidence that essential oils are active against a wide range of microorganisms, including bacteria, fungi, parasites, and viruses, with the ability to eliminate pathogens while preserving “friendly” microbiota [3,5,13,14].

However, good antimicrobial activity alone is insufficient from a clinical point of view. In fact, clinical experience shows that the therapeutic efficacy of conventional antimicrobial agents is dependent on both their direct effect on a given microorganism and the host immune system’s activity [15,16,17,18,19,20]. As with conventional drugs, in order to eradicate the infectious agent, it is critical to assess the potential influence of essential oils on host defense mechanisms in order to identify compounds that can stimulate them rather than interfere negatively with them [15,16,17,18,19,20]. Here, we summarize the current findings of our recent studies about one aspect of the innate immune system, i.e., the interaction of some essential oils on the primary functions of human polymorphonuclear cells (PMNs) from human healthy subjects against some fungal clinical pathogens. PMNs are important effectors of the innate immune system, representing the first line of defense along with monocyte cells, macrophages, dendritic cells, etc. [15,21]. The effects of thyme red oil (26.52% thymol chemotype), *Melaleuca alternifolia* essential oil (tea tree oil, TTO), and *Mentha x piperita* (Huds) var. *officinalis* (Sole), form *rubescens* (Camus) (*Lamiaceae*) essential oils on the killing of intra-PMN yeasts (*Candida albicans* and *C. krusei*) are reported and compared with conventional drugs to determine how they affect the microbicidal activity of human phagocytes in the context of a potential infection. In addition, our results have been compared with the literature data on essential oils and immune system interactions. This comparison would help to fill in any remaining gaps in the understanding of essential oils bioactivity and would help to identify therapeutic solutions to the increasingly common antibiotic resistance.

## 2. Studies Research

Our research group has been investigating for years the in vitro activity of antimicrobial drugs against clinical and environmental bacteria and fungi strains (yeasts and filamentous fungi) [22,23,24,25,26,27,28], as well as the impact of these drugs on the innate immune system of healthy and immunocompromised hosts (renal transplant recipients and hemodialyzed patients) [29,30,31,32,33].

To complete our studies, we compared the antifungal activity of many essential oils (e.g., pine, thyme, *Coridothymus capitatus* and its hydrolate, clove, lavender, lemon balm, sage, fennel, tea tree oil (TTO), *Mentha x piperita* of Pancalieri-MPP, and others) and/or their components (thymol, carvacrol, eugenol, menthol, and others) with antimicrobial drugs against yeasts (*Candida* spp. and non-*Candida* spp.) and filamentous fungi using the same scientific approach used for conventional drugs [7,8,34,35,36].

To make a valid assessment of essential oils’ antimicrobial activity and an effective comparison with conventional drugs, classic microbiological parameters such as the minimal inhibitory concentration (MIC), the bactericide/fungicide concentration (MBC/MFC), the post-antibiotic effect (PAE), the antibiogram/aromatogram, and so on, must be examined, following the international and approved guidelines for conventional drugs (i.e., CLSI and/or EUCAST) [28,37,38], adapting them to essential oils [34,35,36]. Today, we are aware that essential oils have strong antimicrobial properties, even if each oil has a specific microorganism that it inhibits, and not all essential oils may have excellent activity on all microorganisms [7,8,35].

**Figure 1:**
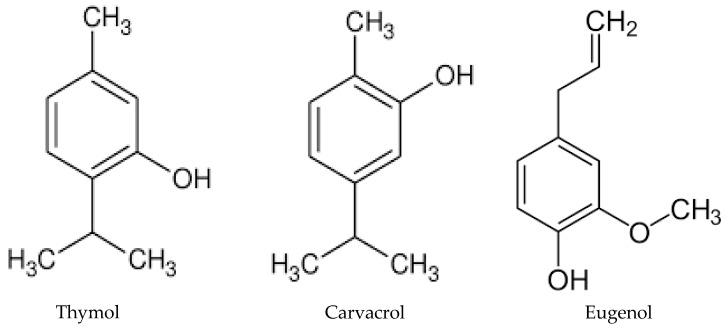
Chemical structures of thymol, carvacrol, and eugenol, the main components of thyme, oregano, and clove essential oils, respectively.

Our data show the real efficacy of essential oils, and in particular, the ability of some of them to inhibit the growth of yeasts and filamentous fungi (dermatophytes, *Fusarium*), already at low concentrations [7]. This is the case of the essential oils of *Thymus vulgaris* (thyme red), pine, clove, oregano, TTO, and MPP, which are proven to be the most effective, showing MIC values that are, in some cases, very low, especially towards the strains of *Cryptococcus neoformans, C. albicans*, *C. glabrata,* and *C. krusei* [33,34,35]. These findings are of great importance in light of the evident resistance that many strains of *C. glabrata* show against traditional therapies with azoles, and the fact that *C. krusei* is naturally resistant to fluconazole. It is not surprising that thyme, oregano, and clove essential oils exhibit a high inhibition of fungal growth, as their main components are phenols, such as thymol (26.52% *v*/*v*), carvacrol (62.61% *v*/*v*), and eugenol (77.5% *v*/*v*), compounds characterized by high bioavailability. These components may determine yeast membrane deterioration through oxidative stress and alteration of the antioxidant defense system even at low concentrations, leading to altered yeast virulence (Figure 1) [8,35].

**Figure 2:**
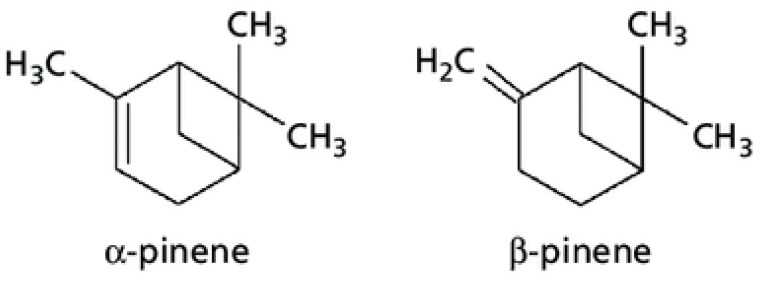
Chemical structures of α- and β-pinene.

Pine essential oil, surprisingly, show, for some strains of non-*albicans Candida* (*C. krusei*, *C. norvegensis*, *C. lusitaniae*, *C. valida*) and uncommon yeasts (*C. neoformans*, *Pichia etchellsii*/*carsonii*, *Saccharomyces cerevisiae*) higher inhibiting activity than thyme, clove, and oregano. Its major components are terpene hydrocarbons, such as α-pinene (55.76% *v*/*v*) and β-pinene (9.034% *v*/*v*) [8] which, as many studies have already shown, possess high antimicrobial abilities, not only against fungi, but also against bacteria and viruses, proving to be one of the most active classes of compounds of plant origin (Figure 2) [8,34,35,39,40,41].

Finally, essential oils that have demonstrated good antimicrobial activity in vitro have been studied to evaluate their influence on the innate defensive mechanisms.

Since 2011, the effects of thyme red oil (thymol chemotype), one of the most active oils from a microbiological point of view, were investigated at subinhibitory and inhibitory concentrations to see how it may influence the intracellular killing activity by human PMNs against *C. albicans* (thyme sensitive) [42,43] and *C. krusei* [44] naturally resistant to fluconazole, in comparison with conventional antifungal drugs (Table 1 and Table 2).

For intracellular studies, human PMNs from healthy subjects were infected with yeast cells and treated with the essential oil at 1/2 × MIC and 1 × MIC. Intracellular killing values were expressed as the yeast survival index (SI) after 30, 60, and 90 min of incubation. Essential-oil-free controls were also included. In addition, in order to provide a frame of reference for the activity of this oil, its time–kill curve without PMNs was also evaluated. Extracellular studies in broth were performed using thyme red oil from 0.1 to 2 × MIC. Viable yeasts were enumerated at 0, 30, 60, 90, 120, 180 min, and 24 h [42]. The influence of the essential oil on intracellular and extracellular activity was compared with that of fluconazole, caspofungin, and anidulafungin, some of the most widely used drugs in the prophylaxis and treatment of candidiasis [32,45].

When compared to essential-oil-free controls, thyme red oil significantly stimulates the killing of intracellular *C. albicans* (Table 1) and *C. krusei* (Table 2) by PMNs, even at subinhibitory concentrations (1/2 MIC), with killing rates overlapping (for *C. albicans*; Table 1) or significantly higher (for *C. krusei*; Table 2) than those observed with fluconazole and anidulafungin. Caspofungin-containing systems further inhibit the survival of *C. albicans*, with fungicidal activity higher, both at 1/2 × MIC and 1 × MIC, than that of essential oil and fluconazole (Table 1) [42,43,44].

In the absence of PMNs, the activity of essential oil against *C. albicans* and *C. krusei* is only fungistatic but greater than it is in the presence of fluconazole and/or anidulafungin [42,44]. In fact, even at 1/2 MIC, oil kills *C. krusei* more efficiently than anidulafungin does in the same experimental conditions at higher concentrations (Table 3). The fact that thyme red oil only has fungistatic activity suggests that the increased fungicidal intracellular killing by PMNs is the result of thyme red oil and phagocytes interacting favorably, similar to what is observed with conventional drugs [29,32,33]. The mechanism of intracellular killing enhancement by oil is still unknown, even if a possible synergistic antifungal interaction with human phagocytes could help to explain why this essential oil, which appears to be only fungistatic in time–kill assays, has great efficacy in killing yeast cells once simultaneously incubated with PMNs.

Although thyme red oil is regarded as one of the best essential oils with antimicrobial activity and has been extensively studied against bacteria, fungi, and viruses, data in the literature about its influence on the immune system is scant in fungal infections and mostly relates to its anti-inflammatory activity observed in mouse models or using various human and murine cell lines [46,47,48,49]. The major components of thyme red oil may contribute to their effects on phagocytes macrophages. Thymol, carvacrol, and linalool possess anti-inflammatory effects by reducing IL-6, IL-1beta, and/or TNF-alfa via the downregulation of the NFkB pathway [45]. The recent study by Harvath et al. [46] suggested a potential use of this oil in neuroinflammation by demonstrating how three different chemotypes of thyme red oil and their primary constituents affect microglia’s production of pro-inflammatory IL-6 and TNF cytokines. *T. vulgaris* is not the only thyme species to have anti-inflammatory properties. Zuzarte et al. [48] looked into the anti-inflammatory potential of essential oils of *T. carnosus* and *T. camphoratus*, two different species of *Thymus*. The main components of these thyme oils were cineol and borneol, which inhibited both the expression of the pro-inflammatory proteins iNOX and COX-2, as well as NO production in LPS-stimulated macrophages.

Additional studies suggest that feeding non-ruminant animals with thyme red oil can help prevent bacterial or viral infections. For example, giving broiler chickens 200 mg/kg of essential oils as a supplement increased lymphocyte proliferation, phagocytosis, serum levels of IgG, IgA, and IgM antibodies, and some complement factors against some viral infections [50]. Additionally, supplementing rainbow trout with thyme red oil at a rate of 0.5 mL/kg increased CD4+ and the expression of the C3 complement component, protecting septicemia caused by the bacterium *Aeromonas hidrophila* [51]. The presence of thymol and carvacrol in the essential oil is most likely responsible for this improvement [52,53,54,55].

Lastly, we tested the effects of other essential oils, such as tea tree oil, *Melaleuca alternifolia* (TTO) and *Mentha x piperita* of Pancalieri-MPP (Torino, Italy), at sub-MIC (1/4 and 1/8) concentrations, on human PMNs’ capacity to kill a clinical strain of *C. krusei* resistant to both fluconazole and anidulafungin [56]. Anidulafungin was used as a comparison to assess the killing potential of these essential oils. The viability of intracellular yeasts is significantly reduced by both TTO and MPP at 1/4 × MIC (42–50% vs. 46–61%) and 1/8xMIC (55–62% vs. 49–67%), with killing percentages 2–3 times higher than in controls (22–33%) and systems containing anidulafungin at 1/2 × MIC (27–33%). However, both oils at 1/8 × MIC significantly enhance the intracellular killing of *C. krusei* by PMNs, with killing values comparable or higher than those observed at higher concentrations (1/4 × MIC). This is probably due to the fact that TTO and MPP at 1/4 × MIC are toxic, and they could interfere with the PMN functionality and killing capacity. On the contrary, the presence of 1/8 × MIC of both oils stimulates yeast killing by PMNs, indicating that the decreasing concentrations do not cause lower candidacidal activity.

**Figure 3:**
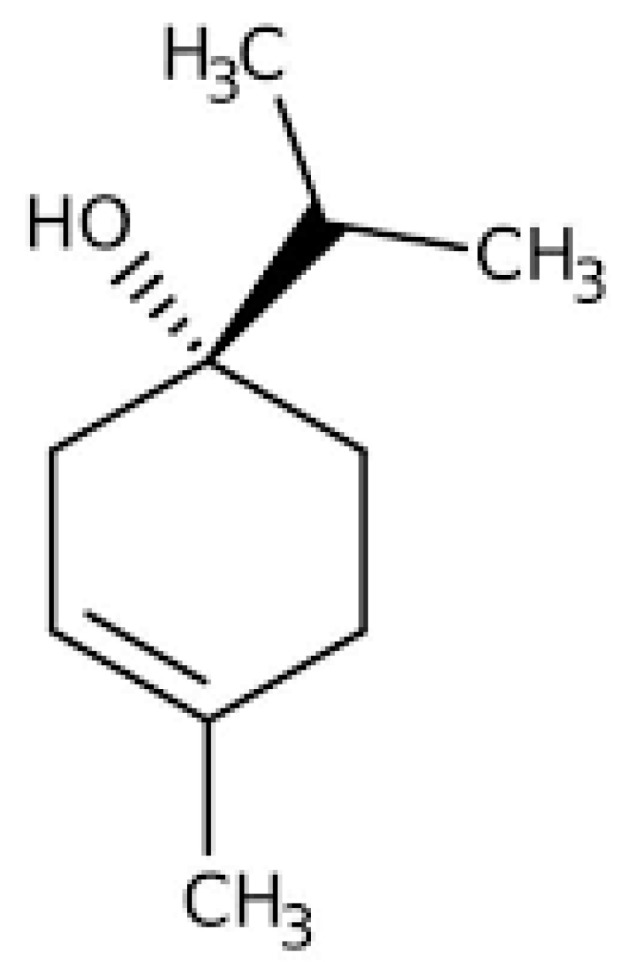
Chemical structure of terpinen-4-ol.

**Figure 4:**
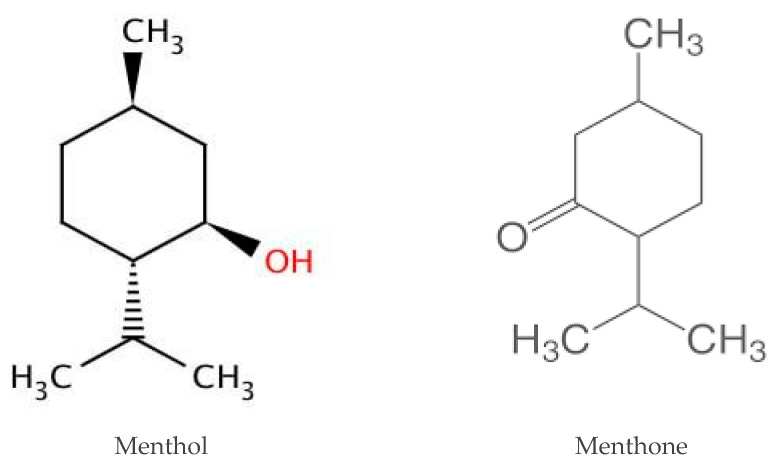
Chemical structure of menthol and menthone.

To highlight whether the action of the oils was directed more toward yeast or phagocytes, or both, the intracellular killing of yeast cells was evaluated after pre-treating the yeast cells and/or phagocytes for 60 min with the essential oils before putting them in contact with PMNs or yeast cells, respectively. The fungicidal activity of PMNs was determined after the withdrawal of the essential oils. Results of pre-treatment with essential oils indicate that TTO acts mainly on yeasts and does not interfere with phagocytic cell functions, while MPP acts both on yeasts and on phagocytes, suggesting, as with thyme red oil, a positive interaction between TTO, MPP, and PMNs [56]. The increased intracellular killing that is seen in the presence of thyme red oil, TTO, and MPP may be the result of direct damage to the yeast cell by essential oils, which may be, at least in part, what causes the alterations that render the yeast more vulnerable to PMN lytic processes [57]. In fact, the main components of thyme red oil are thymol and/or carvacrol [7,8,49], while the main component of TTO is terpinen-4-ol (35.88% *v*/*v*) (Figure 3) [56], and the main components of MPP are menthol (41.7% *v*/*v*) and menthone (21.8% *v*/*v*) (Figure 4), which are known to affect membrane integrity and/or alter the biosynthesis of fungal ergosterol. [8,56,57,58,59].

As the effects of TTO and MPP on phagocyte primary function by other authors were not investigated in the presence of *C. albicans* and *C. krusei*, our data for these essential oils are also difficult to compare. Additionally, MPP is a particular variety of *Mentha* grown in Pancalieri in a restricted area of the Piemonte Region near Turin, and its essential oil is produced locally and not on a large scale. However, some studies reveal that TTO has antioxidant activity and is a modulator of the inflammatory/non-specific immune response. In fact, it decreases nitric oxide and reactive oxygen species (ROS) production by leukocytes and lymphocyte proliferation and improves the secretion of superoxide dismutase and anti-inflammatory cytokines, such as IL-10 and IL-4, at a concentration of 0.01 and 0.1%, respectively [60,61,62]. We are not aware of any published research on the immune system effects of *Mentha x piperita* essential oil; however, this mint shows higher antioxidant activity than other *Mentha* spp. [63,64,65]. A study by Karimian et al. [66] demonstrates that another mint species, *M. longifolia*, likely reduces NO secretion in macrophages by inhibiting iNOS mRNA expression and also decreases the expression of pro-inflammatory cytokines such as TNF, thus, showing its utility in inflammatory disease processes. The most likely cause of this biological activity is menthone; in fact, a recent study in mice with artificially induced arthritis found that menthone significantly reduced the release of cytokines that promote inflammation, including TNF-, IL-1, and IL-6 [67].

## 3. Conclusions

The immunomodulating action of a natural product or an essential oil is often mentioned in commercial products, but the question that arises spontaneously in this regard is whether this claimed immunomodulatory action is real, along with the nature of the examined immunity and the methods used to assess it. This is due to the immune system’s complexity, which makes it extremely difficult and time-consuming to examine all of its aspects. In fact, a general statement on the immunomodulatory activity is problematic since the immune system is made up of numerous components and cells that interact with one another to either up- or down-regulate specific functions.

According to published research, essential oils and their components, such as terpenes, and/or phenols, possess anti-inflammatory and antioxidant activities that interest the food, cosmetic, and human health fields [68]. *Origanum vulgare* L. and *Lavandula angustifolia* Mill. stand out as some essential oils with anti-inflammatory activity that inhibits the production of TNF-alpha, IL-6, IL-12, and prostaglandins but stimulates the production of IL-10 and IL-4 [52,69]. *Echinacea purpurea* (L.) Moench (*Asteraceae*) essential oil shows anti-inflammatory effects in animal models, such as mice and rats, reducing pro-inflammatory citokines such as IL-2, IL-6, and TNF-alfa in the blood [70].

Many other essential oils, including those from *Astragalus membranaceous* (Fisch. Ex Link) Bge (*Fabaceae*) [71], and from spice used in the Mediterranean diet such as *Syzygium aromaticum* (L.) Merr. & L.M.Perry, 1939, *Origanum vulgare* L., *Ocimum basilicum* L., *Salvia officinalis* L., and *Rosmarinus officinalis* L. exhibit antioxidant activity by producing less nitric oxide and reactive oxygen species and more superoxide dismutase [68,72].

With respect to the influence of essential oils on immune functions parameters, it is demonstrated that some essential oils may increase the number of immunocompetent cells, including PMNs, macrophages, dendritic cells, natural killer cells, and B and T lymphocytes [52,73]. *Allium sativum* essential oil and some of its organosulfur components are shown to have a positive effect on macrophage phagocytosis and can activate macrophage chemotaxis, human neutrophil responses with ROS production, and lymphocyte proliferation [74,75]. *Eucalyptus* essential oil also shows the same properties of stimulating phagocytosis by macrophages [76,77]. On the other hand, “Roman coriander” (*Nigella sativa* L., *Ranunculaceae*) essential oil has not been found to have any beneficial or detrimental effects on neutrophil activities [78]. However, recent research shows that thymoquinone, the primary component of the seed essential oil of *N.sativa*, has the ability to inhibit in vitro the proliferation of CD4+ and CD8+ lymphocytes and to induce apoptosis in a dose-dependent manner [79]. A recent evaluation of the biological activities of *Juniperus* spp. essential oils and their main component cedrol shows that they induce intracellular Ca^2+^ mobilization in human neutrophils, which is a key component of neutrophil activation. Likewise, *Rhododendron albiflorum* essential oils can induce human neutrophil and microglial cell Ca^2+^ influx, which desensitizes these cells to subsequent agonist-induced functional responses. A reduction in Ca^2+^ reduces the phagocytic capacity of neutrophils and mononuclear cell response to antigens [80,81].

The results are incomplete and lacking because the research is concerned with only a few pieces of a larger “puzzle” that needs to be completed, even though the amount of data in the literature is actually increasing. To this “puzzle”, we can also add the results of our studies on thyme red oil, TTO, and *Mentha x piperita* of Pancalieri, which show how these oils have a positive impact on some human phagocyte functions. As published data on the intracellular killing of yeast cells by PMNs in the presence of essential oils are extremely scarce and do not address the activity of essential oils in comparison to antifungal drugs, our results may contribute to our understanding of this vast field, even though these findings are minor components of the complex immune system.

The use of essential oils is intriguing, and data from the literature and from our research confirm their good antimicrobial properties and their immunomodulatory activity, which is, in part, comparable to or superior to conventional drugs. Appropriately controlled and randomized clinical investigations, including various screening methods and studies on the mechanisms of action, are encouraged. The therapeutic use of essential oils must be accompanied by a valid scientific investigation that yields results that confirm the suggested therapeutic activity [82,83,84,85]. We must remember that, even if essential oils have fewer side effects, they are not harmless if used incorrectly. In general, investigations show that essential oils do not produce a cytotoxic effect when they are used at low concentrations [56,86]. However, promising clinical microbiology data are frequently insufficient and not based on validated methodologies and experimental models with high predictability for clinical use. In this context, it is critical to implement standardized methods for the in vitro and in vivo evaluation of essential oil activities, just as it is for conventional drugs. It is preferable that various experimental and clinical studies continue to improve the directions, methods, and limitations of these therapeutic agents in order to broaden the range of their potential applications in an effort to develop more effective treatment options in the future.

## Figures and Tables

**Table 1:** Effect of thyme red essential oil, fluconazole, and caspofungin intracellular killing of *Candida albicans* by PMNs.

Survival Index ± SEM							
Time (min)	Controls	Thyme 1/2 × MIC 0.25% (*v*/*v*)	Thyme 1 × MIC 0. 5% (*v*/*v*)	Fluconazole 1/2 × MIC 4 µg/mL	Fluconazole 1 × MIC 8 µg/mL	Calponin 1/2 × MIC 1 µg/mL	Caspofungin 1 × MIC 2 µg/mL
30	1.67 ± 0.03 (33%) ^c^	1.56 ^b^ ± 0.07 (44%)	1.50 ^b^ ± 0.01 (50%)	1.58 ^b^ ± 0.08 (42%)	1.49 ^a^ ± 0.07 (51%)	1.39 ^a^ ± 0.05 (61%)	1.29 ^a^ ± 0.07 (71%)
60	1.53 ± 0.02 (47%)	1.43 ^b^ ± 0.11 (57%)	1.36 ^a^ ± 0.04 (64%)	1.42 ^b^ ± 0.08 (58%)	1.31 ^a^ ± 0.06 (69%)	1.35 ^a^ ± 0.09 (65%)	1.28 ^a^ ± 0.11 (72%)
90	1.50 ± 0.03 (50%)	1.31 ^b^ ± 0.07 (69%)	1.27 ^a^ ± 0.02 (73%)	1.37 ^b^ ± 0.17 (63%)	1.25 ^a^ ± 0.05 (75%)	1.34 ^b^ ± 0.08 (66%)	1.25 ^a^ ± 0.07 (75%)

^a^ Significantly different from the controls (*p* < 0.01); ^b^ significantly different from the controls (*p* < 0.05). ^c^ % Percentages of initial fungal population killed by PMNs in absence/presence of the essential oil and/or drug.

**Table 2:** Effect of thyme red essential oil and anidulafungin on intracellular killing of *Candida krusei* by PMNs.

Survival Index ± SEM			
Time (min)	Controls	Thyme Red Oil 1/2 × MIC 0.25% (*v*/*v*)	Anidulafungin 1/2 × MIC 4 µg/mL
30	1.70 ± 0.03 (30%) ^c^	1.44 ^a^ ± 0.06 (56%)	1.78 ± 0.16 (22%)
60	1.65 ± 0.02 (35%)	1.50 ^a^ ± 0.06 (50%)	1.71 ± 0.01 (29%)
90	1.80 ± 0.03 (20%)	1.63 ^b^ ± 0.03 (37%)	1.67 ^b^ ± 0.15 (33%)

^a^ Significantly different from the controls (*p* < 0.01); ^b^ significantly different from the controls (*p* < 0.05). ^c^ % Percentages of initial fungal population killed by PMNs in absence/presence of the essential oil and/or drug.

**Table 3:** Effect of thyme red essential oil and anidulafungin on the in vitro killing of *Candida krusei* in the absence of PMNs. *C. krusei* (10^6^ cfu/mL) was incubated (Time 0) with the essential oil at 0.5% (1 × MIC) and 0.25% (1/2 × MIC) and/or with anidulafungin at 1 µg/mL (1/8 × MIC), 4 µg/mL (1/2 × MIC), 8 µg/mL (1 × MIC), and 16 µg/mL (2 × MIC) for 0.5, 1, 1.5, 2, 3, and 24 h. Survival yeasts were counted and reported as cfu/mL ± SEM of three separate counts at the end of each incubation time.

CFU/mL ± SEM							
Time (h)	Controls	Thyme Red Oil 1/2 × MIC 0.25% (*v*/*v*)	Thyme red Oil MIC 0.5% (*v*/*v*)	Anidulafungin 1/8 × MIC 1 µg/mL	Anidulafungin 1/2 × MIC 4 µg/mL	Anidulafungin MIC 8 µg/mL	Anidula- fungin 2 × MIC 16 µg/mL
0	1.39 × 10^6^ ± 0.02	2.9 × 10^6^ ± 0.01	5.6 × 10^6^ ± 0.01	3.88 × 10^6^ ± 0.01	1.61 × 10^6^ ± 0.03	6.11 × 10^6^ ± 0.02	4.55 × 10^6^ ± 0.04
0.5	3.36 × 10^6^ ± 0.01	1.55 × 10^4^ ± 0.01	2.1 × 10^4^ ± 0.02	5.55 × 10^6^ ± 0.01	2.25 × 10^6^ ± 0.01	6.83 × 10^6^ ± 0.01	6.24 × 10^6^ ± 0.01
1	1.26 × 10^6^ ± 0.03	2.00 × 10^4^ ± 0.01	1.1 × 10^4^ ± 0.01	3.09 × 10^6^ ± 0.04	1.54 × 10^6^ ± 0.03	2.82 × 10^6^ ± 0.02	4.71 × 10^6^ ± 0.05
1.5	1.53 × 10^6^ ± 0.01	1.00 × 10^4^ ± 0.02	1.5 × 10^4^ ± 0.01	3.43 × 10^6^ ± 0.01	1.69 × 10^6^ ± 0.01	5.22 × 10^6^ ± 0.09	6.50 × 10^6^ ± 0.06
2	5.80 × 10^5^ ± 0.03	6.00 × 10^3^ ± 0.04	1.00 × 10^4^ ± 0.01	3.95 × 10^6^ ± 0.02	1.54 × 10^6^ ± 0.01	2.23 × 10^6^ ± 0.01	3.41 × 10^6^ ± 0.02
3	1.27 × 10^6^ ± 0.01	2.00 × 10^4^ ± 0.01	0	5.55 × 10^6^ ± 0.06	4.06 × 10^6^ ± 0.02	6.53 × 10^6^ ± 0.03	2.6 × 10^6^ ± 0.01
24	4.73 × 10^7^ ± 0.03	0	0	1.60 × 10^7^ ± 0.01	1.10 × 10^6^ ± 0.01	5.15 × 10^6^ ± 0.02	1.21 × 10^7^ ± 0.01

## Data Availability

The data presented in this review are available on request from the first author Vivian Tullio. The data are not publicly available due to privacy.

## Sample Availability

Samples of all the compounds and fungal strains are available from the authors.
